# Obesity-and lipid-related indices as a risk factor of hypertension in mid-aged and elderly Chinese: a cross-sectional study

**DOI:** 10.1186/s12877-023-04650-2

**Published:** 2024-01-20

**Authors:** Jiaofeng Gui, Yuqing Li, Haiyang Liu, Lei-lei Guo, Jinlong Li, Yunxiao Lei, Xiaoping Li, Lu Sun, Liu Yang, Ting Yuan, Congzhi Wang, Dongmei Zhang, Jing Li, Mingming Liu, Ying Hua, Lin Zhang

**Affiliations:** 1 Department of Graduate School, Wannan Medical College, Wuhu, Anhui China; 2 Student Health Center, Wannan Medical College, Wuhu, Anhui China; 3 Department of Surgical Nursing, School of Nursing, Jinzhou Medical University, Jinzhou, Liaoning China; 4 Department of Occupational and Environmental Health, Key Laboratory of Occupational Health and Safety for Coal Industry in Hebei Province, School of Public Health, North China University of Science and Technology, Tangshan, Hebei China; 5 Obstetrics and Gynecology Nursing, School of Nursing, Wannan Medical College, Wuhu, Anhui China; 6 Department of Emergency and Critical Care Nursing, School of Nursing, Wannan Medical College, Wuhu, Anhui China; 7 Department of Internal Medicine Nursing, School of Nursing, Wannan Medical College, Wuhu, Anhui China; 8 Department of Pediatric Nursing, School of Nursing, Wannan Medical College, Wuhu, Anhui China; 9 Department of Surgical Nursing, School of Nursing, Wannan Medical College, Wuhu, Anhui China; 10 Rehabilitation Nursing, School of Nursing, Wanna Medical College, Wuhu, Anhui China

**Keywords:** Hypertension, Lipids, Obesity, Middle-aged and elderly Chinese

## Abstract

**Objective:**

Hypertension refers to the persistent elevation of blood pressure above the established normal range, resulting in increased pressure exerted by blood on the walls of blood vessels during its circulation. Recent studies have identified significant associations between obesity and lipid-related indices, as well as hypertension. Nevertheless, these studies have yet to comprehensively examine the correlation between the two variables. Our objective is to identify the fat and lipid-related indices that have the strongest correlation with hypertension.

**Method:**

There was a total of 9488 elderly and middle-aged Chinese citizens who participated in this investigation. The participants in this research were separated into distinct gender cohorts. The participants were classified into normal and hypertensive categories according to their gender, with hypertension defined as a blood pressure level of 140/90 mmHg or higher, or a history of hypertension. Through the utilization of binary logistic regression analyses and the receiver operator curve (ROC), the optimal among fourteen indicators associated with obesity and lipids were identified.

**Results:**

After adjusting for variables, statistical analysis showed that all 14 measures of obesity and lipid were risk factors for hypertension. The receiver operating characteristic (ROC) curve analysis reveals that the Chinese visceral adiposity index (CVAI) has the highest degree of relationship to hypertension. Simultaneously, a statistically significant association between hypertension and these 14 variables was observed in both males and females.

**Conclusion:**

There was a significant independent association between various parameters related to obesity and lipid-related index and the presence of hypertension, indicating that these factors can be considered risk factors for hypertension. CVAI and WHtR (waist height ratio) can be used to screen the high-risk groups of hypertensions in middle-aged and elderly people in China, and then take individualized health care measures to reduce the harm of hypertension.

**Supplementary Information:**

The online version contains supplementary material available at 10.1186/s12877-023-04650-2.

## Introduction

Hypertension stands as the prevailing risk factor for cardiovascular disease within the middle-aged and elderly population of China. Hypertension is defined as systolic blood pressure (SBP) ≥ 140mmhg, and/or diastolic blood pressure ≤ 90mmhg, and/or taking antihypertensive drugs within 2 weeks [1]. According to a national survey on high blood pressure among adults in China conducted since 2012, the prevalence of high blood pressure among adults in China is about 23.2% [2]. With the aging of the population and increasing obesity, the number of people with high blood pressure has increased, and it is estimated that one-third of the world’s population will suffer from high blood pressure by 2025 [3].

Hypertension is the most common risk factor for heart disease and a major risk factor for death and disability worldwide [4]. The decline of the metabolic ability of middle-aged and elderly people is accompanied by various basic diseases, which makes hypertension cause a greater risk of disability and death. Therefore, this study aims to find a more effective index to predict the occurrence of hypertension. Population and social development factors (age, gender, education, and region) are closely related to the prevalence of hypertension in China middle-aged and elderly people [5–7]. Smoking and drinking are significantly related to the incidence of hypertension [8]. At the same time, You, Y [9] research shows that strenuous physical activity does a good job of preventing high blood pressure.

People with metabolic disorders, such as insulin resistance, are more likely to have hypertension than those without [10]. Insulin resistance often leads to compensatory hyperinsulinemia, and the elevated insulin in the blood will affect the body’s renal sodium excretion and sympathetic nerve activity. This persistent effect may lead to the development of hypertension [11]. At the same time, there is a significant relationship between SUA (serum uric acid) and the risk of hypertension [12]. At present, the most recognized mechanism is that SUA induces inflammation of vascular endothelial cells and smooth muscle cells, as well as oxidative stress within cells, leading to endothelial dysfunction [13]. This long-term functional impairment may be an important reason for the occurrence of hypertension.

Obesity and lipid indicators have been widely used in the Chinese population. Studies have confirmed that the triglyceride glucose index (TyG-index) can be used as a reliable and convenient indicator to assess insulin resistance, and can also be optimized for risk stratification and prediction of CVD (cardiovascular disease) [14]. Lipid indicators are closely associated with carotid intima-media thickness in young adults, and this association can be used to predict subclinical atherosclerosis [15]. A large body of clinical and epidemiological evidence suggests a close relationship between obesity and hypertension [16]. Obese individuals are often accompanied by lipid accumulation in the body. Therefore, obesity and lipid indicators have become the main predictors of hypertension. It has been confirmed that obesity and lipid-related indices are related to hypertension. At present, the main research direction is the strength of association between each index and hypertension.

Previous studies have shown that obesity and lipid indicators are closely related to hypertension. However, most cross-sectional studies only explore the strength of the association between a single or a few indicators and hypertension, without comparing the advantages of each indicator. At the same time, due to the large differences in diet, culture, religion, and life between China and other countries, relevant studies abroad do not apply to the Chinese population. Therefore, it is necessary to explore the obesity and lipid indicators that are most closely associated with hypertension.

The data of this cross-sectional study were collected from community residents aged 45 years and older across China, and it is a national survey study. At the same time, a total of 14 obesity and lipid indicators were included, and by comparing the advantages of different indicators, we aimed to find the indicator most closely related to hypertension.

## Materials and methods

### Participants

Participants in this cross-sectional study were China community residents over the age of 45. All participants were from the China Health and Retirement Longitudinal Study (CHARLS). CHARLS is a nationally representative longitudinal survey. Every 2 years, CHARLS conducted computer-assisted personal interviews (CAPI) and structured questionnaires with participants. In the survey, 17,284 participants were 45 years of age or older. CHARLS collected data from 2011, 2013, and 2015, and we used the data from 2011. We excluded participants who were not followed up, as well as any standard individuals without data on age, sex, education, smoking history, activity participation, regular exercise, and chronic disease. The number of people who completed both baselines without hypertension symptoms was 9488.

### Hypertension symptom

According to the 2018 Chinese Guidelines for Prevention and Treatment of Hypertension [17], Clinical systolic blood pressure ≥ of 140 mmHg or diastolic blood pressure ≥ of 90 mmHg was defined as hypertension. Blood pressure is usually measured with an international standardized upper arm medical electronic sphygmomanometer or a mercury sphygmomanometer that meets measurement standards (at least 5 min of sitting in a quiet environment). Diagnosis of hypertension is divided into two categories:

1. Clinic systolic BP ≥ 140 mmHg or diastolic BP ≥ 90 mmHg (antihypertensive drugs were never used in three different visits).
2. Clinic systolic BP < 140/90 mmHg (having a hypertensive history and currently taking anti-hypertensive medication).

### Covariates

In this study, we divided the participants into a male group and a female group. At the same time, we regard age, education level, marital status, current residence, current smoking, drinking, chronic diseases, participation in activities, and regular exercise as covariates of this study. We counted 14 chronic diseases and grouped them according to the number of diseases. This study reflected the presence of chronic health conditions by assessing 14 common chronic symptoms in middle-aged and elderly people. The 14 chronic conditions include high blood pressure, dyslipidemia, diabetes or high blood sugar, cancer or malignancy, chronic lung disease, liver disease, heart disease, stroke, kidney disease, digestive system diseases, neurological or psychiatric conditions, memory-related diseases, arthritis or rheumatic diseases, and asthma. Each disease was recorded as 1 point, and the sum of the scores for all diseases (ranging from 0 to 14) was used as a chronic disease indicator. SUA is a continuous variable, and the unit is mg/dL. The remaining nine covariates are shown below.

1. Age: 1) below 45–54, 2) 55–64, 3) 65–74, 4) above 75.
2. Education level: 1) illiterate, 2) less than elementary school,3) high school, 4) above vocational school.
3. Marital status: 1) the single (divorced, and never married, widowed, or separated), 2) married.
4. Current residence: 1) rural, 2) urban.
5. Current smoking: 1) current smokers, 2) former smokers, 3) never smokers.
6. Alcohol drinking: 1) never drinker, 2) less than once a month, 3) more than once a month.
7. Taking activities: 1) yes, 2) no.
8. Having regular exercises: 1) no physical exercise, 2) less than regular physical exercises, 3) regular physical exercises.
9. Chronic diseases: 1) 0, 2) 1–3, 3) 4–14.

### Measurements

Waist circumference (WC) is the circumference of the line connecting the lowest point of the rib to the midpoint of the upper edge of the iliac crest before the end of expiratory breath [18]. It should be noted that the other 13 indicators need to be calculated. At the same time, some indicators need to be invasive to obtain TG and HDL. Body mass index (BMI) is the value of weight (kg) divided by the square of height (m). Waist height ratio (WHtR) is the ratio of WC (m) to height (m). The calculation of visceral adiposity index (VAI) differs between males and females but is based on WC, BMI, TG, and HDL. Chinese visceral adiposity index (CVAI) is based on VAI, according to the physical characteristics of China people to develop indicators. Similarly, the calculation method of lipid accumulation product (LAP) is different due to gender differences. A body shape index (ABSI) is obtained by WC, BMI, and height. Body roundness index (BRI) is obtained through WC and height. The conicity index (CI) is obtained through WC, weight, and height. The triglyceride glucose index (TyG-index) is a lipid index calculated from TG and glucose. At the same time, TyG index combined with BMI, WC, and WHtR constitutes TyG-BMI, TyG-WC, and TyG-WHtR. METS-IR (metabolic score for insulin resistance) is an indicator of metabolic function in healthy or high-risk populations, and it is also an effective tool for screening insulin resistance. We have listed the calculation formula for the 13 indicators below:

1. BMI = Weight/Height^2^ [19]
2. WHtR = WC/Height [20]
3. Males: $${\text{VAI}}=\mathrm{ WC}/(1.88\times {\text{BMI}}+39.68)\times {\text{TG}}/1.03\times 1.31/{\text{HDL-C}}$$

Females: $${\text{VAI}}={\text{WC}}/(1.89\times {\text{BMI}}+36.58) \times {\text{TG}}/0.81\times 1.52/{\text{HDL-C}}$$ [21]

(4) $${\text{ABSI}}= \frac{WC}{{H{\text{eight}}}^\frac{1}{2}\times {BMI}^\frac{2}{3}}$$ [22]
(5) $${\text{BRI}}= 364.2-365.5\sqrt{1-\left(\frac{WC\div {\left(2\pi \right)}^{2}}{{\left(0.5\times Height\right)}^{2}}\right)}$$ [23]
(6) Males: $${\text{LAP}}=\mathrm{ TG }\times ({\text{WC}}-65)$$, Females: $${\text{LAP}}=\mathrm{ TG }\times ({\text{WC}}-58)$$ [24]
(7) $${\text{CI}}={\text{WC}}/\sqrt[0.19]{{\text{Weight}}/{\text{Height}}}$$ [25]
(8) Males: $${\text{CVAI}}=-267.93-16.32\times {\text{HDL-C}}+0.03\times {\text{BMI}}+22.00\times {{\text{Log}}}_{10}\text{TG }+0.68\times {\text{age}}+4.00\times {\text{WC}}$$

Females: $${\text{CVAI}}=-187.32-11.66\times {\text{HDL-C}}+4.32\times \text{BMI }+39.76\times {{\text{Log}}}_{10}\text{TG }+1.71\times {\text{age}}+1.12\times {\text{WC}}$$ [26]

(9) TyG index = Ln (TG × glucose/2) [27]
(10) $${\text{TyG-BMI}}=\text{TyG}\times{\text{BMI}},\text{TyG-WC}=\text{TyG}\times {\text{WC}},\text{ TyG-WHtR}=\text{ TyG}\times {\text{WHtR}}$$ [28, 29]
(11) METS-IR = Ln[(2* glucose) + TG]*BMI)/(Ln[HDL-C]) [30]

The 14 indicators selected in this study can be obtained by simple body measurements and simple blood chemistry tests.

### Statistical analysis

In this study, SPSS version 25.0 (IBM SPSS, Armonk, NY, USA) was used for statistical analysis of all obtained sample data. The chi-square test was applied to classified variables and the t-test was applied to continuous variables to determine the strength of differences between the variables. The odds ratio (OR) of obesity-related and lipid-related indices to hypertension before and after adjustment of the covariates (Supplemental Table 1) was calculated. These covariates included age, education level, marital status, current residence, current smoking, alcohol drinking, taking activities, regular exercise, chronic diseases, and SUA. The ability of these indicators to assess hypertension was compared by receiver operator curve (ROC).

## Results

Table 1 shows the baseline characteristics of participants according to gender differences. The number of participants was 9488. 45.89% were male, and 54.11% were female. 12.45% were single. 92.40% were living in rural. 24.69% of males do not smoke, 92.19% of females do not smoke, 13.71% of males have never received an education, and 42.56% of females have never received an education. Among them, 24.69% of males have never smoked, and 92.19% of females have never smoked. 58.45% of males are smoking, and 5.96% of females are smoking. There are significant differences in education level, smoking, and drinking between males and females. In addition to METS-IR, the remaining 13 measures of obesity and lipids differed between male and female groups (*P* < 0.05).

**Table 1:** Characteristics of participants with full samples (*N* = 9488)

**Variables**	**Male**	**Female**	***P***
	**N (%)**	**N (%)**	
N	4354 (100)	5134 (100)	
Age (years)			0.000
45–54	1274 (29.26)	1922 (37.44)	
55–64	1724 (39.60)	1930 (37.59)	
65–74	989 (22.71)	903 (17.59)	
≥ 75	367 (8.43)	379 (7.38)	
Education			0.000
Illiterate	597 (13.71)	2185 (42.56)	
Less than elementary school	3195 (73.38)	2604 (50.72)	
High school	360 (8.27)	253 (4.93)	
Above vocational school	202 (4.64)	92 (1.79)	
Marital status			0.000
Single	406 (9.32)	775 (15.10)	
Married	3948 (90.68)	4359 (84.90)	
Current residence			0.387
Rural	4012 (92.15)	4755 (92.62)	
Urban	342 (7.85)	379 (7.38)	
Current smoking			0.000
No	1075 (24.69)	4733 (92.19)	
Former smoke	734 (16.86)	95 (1.85)	
Current smoke	2545 (58.45)	306 (5.96)	
Alcohol drinking			0.000
No	1920 (44.10)	4516 (87.96)	
Less than once a month	470 (10.79)	255 (4.97)	
More than once a month	1964 (45.11)	363 (7.07)	
Taking activities			0.374
No	2138 (49.10)	2568 (50.02)	
Yes	2216 (50.90)	2566 (49.98)	
Having regular exercises			0.563
No exercise	2708 (62.20)	3145 (61.26)	
Less than exercises	814 (18.70)	1001 (19.50)	
Regular exercises	832 (19.11)	988 (19.24)	
Chronic diseases (counts)			0.000
0	1422 (32.66)	1474 (28.71)	
1–2	2161 (49.63)	2623 (51.09)	
3–14	771 (17.71)	1037 (20.20)	
SUA	4.97 ± 1.27	4.01 ± 1.06	0.000
METS-IR	34.8 ± 8.16	36.15 ± 8.11	0.580
WC	84.96 ± 9.81	85.64 ± 10.16	0.001
BMI	22.96 ± 3.64	23.99 ± 4.05	0.000
WHtR	0.52 ± 0.06	0.56 ± 0.07	0.000
VAI	3.96 ± 4.41	6.07 ± 5.72	0.000
ABSI	8.25 ± 0.53	8.38 ± 0.64	0.000
BRI	3.78 ± 1.14	4.66 ± 1.46	0.000
LAP	30.87 ± 33.31	43.74 ± 35.23	0.000
CI	1.27 ± 0.08	1.30 ± 0.10	0.000
CVAI	95.98 ± 47.50	107.11 ± 43.43	0.000
TyG index	8.62 ± 0.66	8.72 ± 0.63	0.000
TyG-BMI	198.67 ± 39.64	209.77 ± 41.67	0.000
TyG-WC	734.60 ± 117.99	748.68 ± 116.22	0.000
TyG -WHtR	4.48 ± 0.69	4.91 ± 0.76	0.000

*SUA* Serum uric acid, *METS-IR* Metabolic score for insulin resistance, *WC* Waist circumference, *BMI* Body mass index, *WHtR* Waist height ratio, *VAI* Visceral adiposity index, *ABSI* A body shape index, *BRI* Body roundness index, *LAP* Lipid accumulation product, *CVAI* Chinese visceral adiposity index, *CI* Conicity index, *TyG* Triglyceride glucose, *TyG-BMI* TyG related to BMI, *TyG-WC* TyG related to WC, *TyG-WHtR* TyG related to WHtR

Table 2 shows the baseline characteristics of men and women with and without hypertension, respectively. 9488 participants were divided into two groups. In 4354 males, 1742(40.01%) males had hypertension and 2612(59.99%) males had no hypertension of 5134 females, 2162(42.11%) females suffered from hypertension, and 2972(57.89%) females did not suffer from hypertension. There are differences in individual characteristics, and SUA between hypertensive patients and non-hypertensive patients. It is worth noting that smoking and alcohol drinking did not show statistical differences among males (*P* > 0.05). There were also differences in 14 indexes between the two groups (*P* < 0.05).

**Table 2:** Baseline characteristics of the study participants with and without HTN by sex

**Variables**	**Male (*N* = 4354)**		***P***	**Female (*N* = 5134)**		***P***
**N (%)**	**With HTN N (%)**	**Without HTN N (%)**		**With HTN N (%)**	**Without HTN N (%)**	
N	1742 (40.01)	2612 (59.99)		2162 (42.11)	2972 (57.89)	
Age (years)			0.000			0.000
45–54	392 (22.50)	882 (33.77)		1389 (53.18)	533 (30.60)	
55–64	686 (39.38)	1038 (39.74)		1085 (41.54)	845 (48.51)	
65–74	471 (27.04)	518 (19.83)		378 (14.47)	525 (30.14)	
≥ 75	193 (11.08)	174 (6.66)		120 (4.59)	259 (14.87)	
Education			0.002			0.001
Illiterate	256 (14.70)	341 (13.06)		1017 (58.38)	1168 (44.72)	
Less than elementary school	1273 (73.08)	1922 (73.58)		1031 (59.18)	1573 (60.22)	
High school	117 (6.72)	243 (9.30)		80 (4.59)	173 (6.62)	
Above vocational school	96 (5.51)	106 (4.06)		34 (1.95)	58 (2.22)	
Marital status			0.000			0.000
Single	205 (11.77)	201 (7.70)		441 (25.32)	334 (12.79)	
Married	1537 (88.23)	2411 (92.30)		1721 (98.79)	2638 (101.00)	
Current residence			0.004			0.948
Rural	1580 (90.70)	2432 (93.11)		2003 (114.98)	2752 (105.36)	
Urban	162 (9.30)	180 (6.89)		159 (9.13)	220 (8.42)	
Current smoking			0.000			0.016
No	448 (25.72)	627 (24.00)		1966 (112.86)	2767 (105.93)	
Former smoke	340 (19.52)	394 (15.08)		48 (2.76)	47 (1.80)	
Current smoke	954 (54.76)	1591 (60.91)		148 (8.50)	158 (6.05)	
Alcohol drinking			0.332			0.010
No	792 (45.46)	1128 (43.19)		1935 (111.08)	2581 (98.81)	
Less than once a month	183 (10.51)	287 (10.99)		88 (5.05)	167 (6.39)	
More than once a month	767 (44.03)	1197 (45.83)		139 (7.98)	224 (8.58)	
Taking activities			0.366			0.034
No	870 (49.94)	1268 (48.55)		1044 (59.93)	1524 (58.35)	
Yes	872 (50.06)	1344 (51.45)		1118 (64.18)	1448 (55.44)	
Having regular exercises			0.486			0.028
No exercise	1091 (62.63)	1617 (61.91)		1368 (78.53)	1777 (68.03)	
Less than exercises	311 (17.85)	503 (19.26)		390 (22.39)	611 (23.39)	
Regular exercises	340 (19.52)	492 (18.84)		404 (23.19)	584 (22.36)	
Chronic diseases (counts)			0.000			0.000
0	344 (19.75)	1078 (41.27)		351 (20.15)	1123 (42.99)	
1–2	898 (51.55)	1263 (48.35)		1106 (63.49)	1517 (58.08)	
3–14	500 (28.70)	271 (10.38)		705 (40.47)	332 (12.71)	
SUA	5.17 ± 1.34	4.84 ± 1.20	0.000	4.21 ± 1.11	3.87 ± 1.00	0.000
METS-IR	37.00 ± 9.00	33.33 ± 7.20	0.000	38.23 ± 8.56	34.65 ± 7.41	0.000
WC	88.04 ± 10.39	82.91 ± 8.83	0.000	88.70 ± 10.37	83.42 ± 9.40	0.000
BMI	23.96 ± 3.98	22.29 ± 3.23	0.000	24.89 ± 4.27	23.33 ± 3.74	0.000
WHtR	0.54 ± 0.06	0.51 ± 0.05	0.000	0.58 ± 0.07	0.54 ± 0.06	0.000
VAI	4.56 ± 4.86	3.56 ± 4.03	0.004	7.05 ± 6.50	5.36 ± 4.96	0.000
ABSI	8.31 ± 0.54	8.21 ± 0.51	0.000	8.49 ± 0.68	8.30 ± 0.59	0.000
BRI	4.15 ± 1.23	3.53 ± 1.01	0.000	5.15 ± 1.55	4.31 ± 1.27	0.000
LAP	38.59 ± 38.65	25.71 ± 28.06	0.000	53.08 ± 39.86	36.94 ± 29.65	0.000
CI	1.29 ± 0.09	1.26 ± 0.08	0.000	1.33 ± 0.10	1.28 ± 0.09	0.000
CVAI	112.11 ± 49.52	85.22 ± 42.86	0.000	124.49 ± 42.03	94.46 ± 39.92	0.000
TyG index	8.74 ± 0.68	8.54 ± 0.63	0.000	8.86 ± 0.65	8.62 ± 0.60	0.000
TyG-BMI	210.27 ± 43.49	190.92 ± 34.77	0.000	220.97 ± 43.92	201.63 ± 37.93	0.000
TyG-WC	771.74 ± 125.33	709.82 ± 105.84	0.000	787.09 ± 119.23	720.73 ± 105.55	0.000
TyG -WHtR	4.70 ± 0.73	4.34 ± 0.62	0.000	5.18 ± 0.78	4.71 ± 0.68	0.000

*SUA* Serum uric acid, *METS-IR* Metabolic score for insulin resistance, *WC* Waist circumference, *BMI* Body mass index, *WHtR* Waist height ratio, *VAI* Visceral adiposity index, *ABSI* A body shape index, *BRI* Body roundness index, *LAP* Lipid accumulation product, *CVAI* Chinese visceral adiposity index, *CI* Conicity index, *TyG* Triglyceride glucose, *TyG-BMI* TyG related to BMI, *TyG-WC* TyG related to WC, *TyG-WHtR* TyG related to WHtR

Table 3 shows that hypertension increased progressively with unit increases in obesity- and lipid-related indices in both sexes. For instance, in males, a unit increase in WC was associated with 1.05-fold increased odds of elevated hypertension (OR: 1.05; 95% CI: 1.04–1.06), and a unit increase in BMI was associated with a 1.13-fold increase in odds of elevated hypertension (OR: 1.13; 95% CI: 1.09–1.18). In females, a unit increase in WC was associated with a 1.03-fold increase in odds of elevated hypertension (OR: 1.03; 95% CI: 1.02–1.04), and a unit increase in BMI was associated with 1.06-fold increased odds of elevated hypertension (OR: 1.06; 95% CI: 1.03–1.10). After adjusting the risk model with SUA as a covariate, 14 indicators still appeared as risk factors for hypertension.

**Table 3:** Associations of obesity- and lipid-related indices with HTN and its components

**HTN**	**WC**	**BMI**	**WHtR**	**VAI**	**ABSI**	**BRI**	**LAP**	**CI**	**CVAI**	**TyG index**	**TyG-BMI**	**TyG-WC**	**TyG -WHtR**	**METS-IR**
**Male**														
Model 1	1.06 (1.05,1.07)^**^	1.15 (1.13,1.17)^**^	17,700.40 (5574.24,56,205.69)^**^	1.05 (1.04,1.07)^**^	1.43 (1.27,1.61)^**^	1.64 (1.55,1.74)^**^	1.01 (1.01,1.02)^**^	98.57 (44.95,216.14)^**^	1.01 (1.01,1.01)^**^	1.59 (1.44,1.74)^**^	1.01 (1.01,1.02)^**^	1.01 (1.00,1.01)^**^	2.23 (2.03,2.45)^**^	1.06 (1.05,1.07)*
Model 2	1.06 (1.05,1.07)^**^	1.18 (1.15,1.21)^**^	13,059.50 (3795.87,44,930.53)^**^	1.06 (1.04,1.08)^**^	1.24 (1.09,1.40)^**^	1.62 (1.52,1.72)^**^	1.01 (1.01,1.02)^**^	44.84 (19.78,101.66)^**^	1.01 (1.01,1.01)^**^	1.62 (1.46,1.79)^**^	1.02 (1.01,1.02)^**^	1.01 (1.00,1.01)^**^	2.24 (2.02,2.48)^**^	1.07 (1.06,1.08)^**^
Model 3	1.06 (1.05,1.07)^**^	1.17 (1.15,1.20)^**^	8447.91 (2429.43,29,376.04)^**^	1.05 (1.03,1.07)^**^	1.22 (1.07,1.38)^*^	1.58 (1.49,1.68)^**^	1.01 (1.01,1.02)^**^	36.36 (15.98,82.72)^**^	1.01 (1.01,1.01)^**^	1.53 (1.38,1.70)^**^	1.02 (1.01,1.02)^**^	1.01 (1.00,1.01)^**^	2.14 (1.93,2.38)^**^	1.07 (1.06,1.08)^**^
**Female**														
Model 1	1.06 (1.05,1.06)^**^	1.11 (1.09,1.13)^**^	10,821.90 (4254.28,27,528.40)^**^	1.06 (1.04,1.07)^**^	1.63 (1.48,1.78)^**^	1.54 (1.48,1.61)^**^	1.01 (1.01,1.02)^**^	134.43 (71.55,252.55)^**^	1.02 (1.02,1.02)^**^	1.82 (1.66,2.00)^**^	1.01 (1.01,1.01)^**^	1.01 (1.01,1.01)^**^	2.40 (2.21,2.61)^**^	1.06 (1.05,1.07)^**^
Model 2	1.05 (1.04,1.06)^**^	1.14 (1.12,1.16)^**^	1804.76 (671.84,4848.12)^**^	1.05 (1.04,1.06)^**^	1.12 (1.01,1.24)	1.42 (1.35,1.48)^**^	1.01 (1.01,1.01)^**^	16.36 (8.21,32.6)^**^	1.01 (1.01,1.02)^**^	1.62 (1.47,1.79)^**^	1.01 (1.01,1.02)^**^	1.01 (1.00,1.01)^**^	2.04 (1.87,2.22)^**^	1.07 (1.06,1.08)^**^
Model 3	1.05 (1.04,1.06)^**^	1.13 (1.11,1.15)^**^	1181.56 (434.54,3212.78)^**^	1.04 (1.03,1.05)^**^	1.12 (1.00,1.24)^*^	1.39 (1.33,1.46)^**^	1.01 (1.01,1.01)^**^	13.72 (6.86,27.45)^**^	1.01 (1.01,1.01)^**^	1.53 (1.38,1.69)^**^	1.01 (1.01,1.01)^**^	1.00 (1.00,1.01)^**^	1.95 (1.79,2.14)^**^	1.06 (1.05,1.07)^**^

Model 1: Unadjusted

Model 2: Adjusting for age, educational levels, marital status, current residence, current smoking, alcohol drinking, taking activities, having regular exercises, chronic diseases

Model 3: Adjusting for age, educational levels, marital status, current residence, current smoking, alcohol drinking, taking activities, having regular exercises, chronic diseases, serum uric acid

*WC* Waist circumference, *BMI* Body mass index, *WHtR* Waist to height ratio, *VAI* Visceral adiposity index, *ABSI* A body shape index, *BRI* Body roundness index, *LAP* Lipid accumulation product, *CVAI* Chinese visceral adiposity index, *CI* Conicity index, *TyG* Triglyceride glucose, *TyG-BMI* TyG related to BMI, *TyG-WC* TyG related to WC, *TyG-WHtR* TyG related to WHtR, *METS-IR* Metabolic score for insulin resistance

^*^P < 0.05, ^*^^*^P < 0.001

**Fig. 1:**
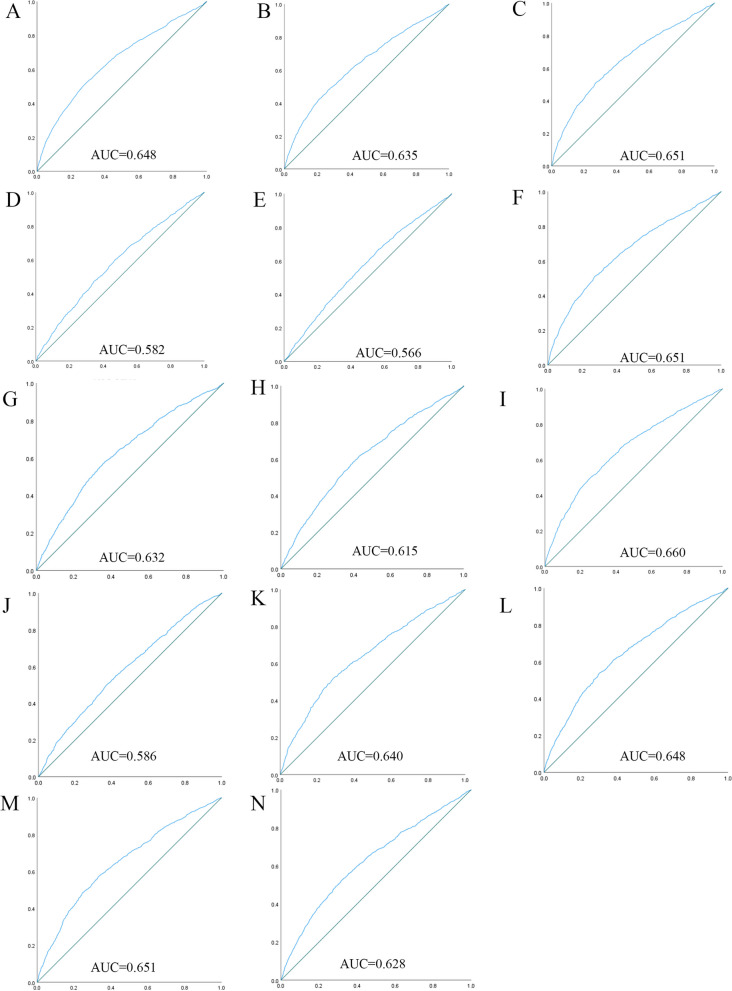
The ROC curves for each indicator and the risk associated with hypertension in males. (**A**) WC, (**B**) BMI, (**C**) WHtR, (**D**) VAI, (**E**) ABSI, (**F**) BRI, (**G**) LAP, (**H**) CI, (**I**) CVAI, (**J**) TyG-index, (**K**) TyG-BMI, (**L**) TyG-WC, (**M**) TyG-WHtR, (**N**) METS-IR

**Fig. 2:**
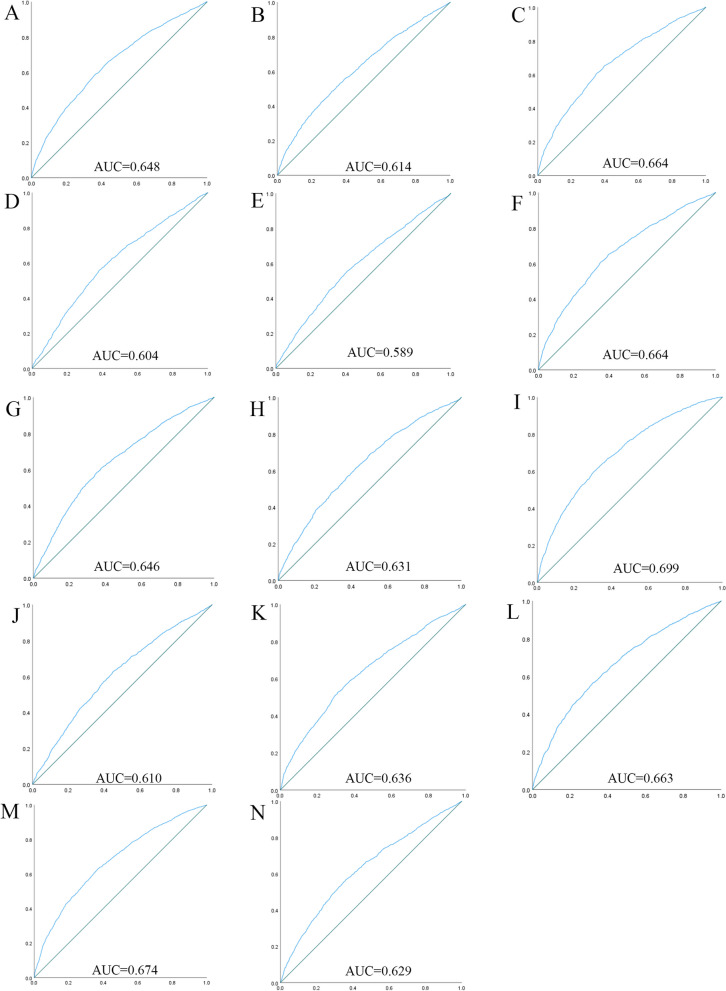
The ROC curves for each indicator and the risk associated with hypertension in females. (**A**) WC, (**B**) BMI, (**C**) WHtR, (**D**) VAI, (**E**) ABSI, (**F**) BRI, (**G**) LAP, (**H**) CI, (**I**) CVAI, (**J**) TyG-index, (**K**) TyG-BMI, (**L**) TyG-WC, (**M**) TyG-WHtR, (**N**) METS-IR

Table 4 shows the ROC analysis between the 13 indicators and hypertension. Figures 1 and 2 show the ROC curve and AUC for males and females, respectively. In males, the largest AUC was observed for the CVAI (AUC = 0.660, 95%CI = 0.643–0.676, and optimal cut-off = 111.142). The relations were similar for the WHtR (AUC = 0.651, 95%CI = 0.635–0.668, and optimal cut-off = 0.534), BRI (AUC = 0.651, 95%CI = 0.635–0.668, and optimal cut-off = 4.013), and TyG -WHtR (AUC = 0.651, 95%CI = 0.634–0.667, and optimal cut-off = 4.525). In women, the largest AUC was CVAI (AUC = 0.699, 95%CI = 0.685–0.713, and optimal cut-off = 113.022). The relations were similar for the TyG -WHtR (AUC = 0.674, 95%CI = 0.660–0.689, and optimal cut-off = 4.924), WHtR (AUC = 0.664, 95%CI = 0.649–0.679, and optimal cut-off = 0.567), and BRI (AUC = 0.664, 95%CI = 0.649–0.679, and optimal cut-off = 4.697). It should be noted that in both sexes, these 14 measures were significantly associated with hypertension (*P* < 0.001).

**Table 4:** Area under curve for obesity-and lipid-related indices to detect HTN by sex

***N* = 4423**	**WC**	**BMI**	**WHtR**	**VAI**	**ABSI**	**BRI**	**LAP**	**CI**	**CVAI**	**TyG index**	**TyG-BMI**	**TyG-WC**	**TyG -WHtR**	**METS-IR**
**Male**														
Area under curve	0.648	0.635	0.651	0.582	0.566	0.651	0.632	0.615	0.660	0.586	0.640	0.648	0.651	0.628
95%CI	0.631,0.665	0.618,0.652	0.635,0.668	0.565,0.600	0.549,0.584	0.635,0.668	0.615,0.649	0.598,0.632	0.643,0.676	0.569,0.603	0.623,0.657	0.631,0.665	0.634,0.667	0.611,0.645
*P*-value	0.000	0.000	0.000	0.000	0.000	0.000	0.000	0.000	0.000	0.000	0.000	0.000	0.000	0.000
Optimal cutoffs	87.250	23.877	0.534	2.516	8.120	4.013	22.955	1.272	111.142	8.647	204.580	750.584	4.525	35.265
J-Youden	0.233	0.212	0.240	0.131	0.106	0.240	0.215	0.189	0.245	0.129	0.234	0.238	0.241	0.206
Sensitivity (%)	51.90	48.10	51.40	59.90	66.90	51.40	57.90	61.50	50.30	49.80	52.20	54.20	57.60	52.40
Specificity (%)	71.40	73.10	72.60	53.20	43.70	72.60	63.60	57.40	74.20	63.10	71.20	69.60	66.50	68.20
**Female**														
Area under curve	0.648	0.614	0.664	0.604	0.589	0.664	0.646	0.631	0.699	0.610	0.636	0.663	0.674	0.629
95%CI	0.633,0.664	0.599,0.630	0.649,0.679	0.588,0.619	0.573,0.604	0.649,0.679	0.631,0.662	0.616,0.646	0.685,0.713	0.594,0.625	0.621,0.651	0.648,0.678	0.660,0.689	0.613,0.644
*P*-value	0.000	0.000	0.000	0.000	0.000	0.000	0.000	0.000	0.000	0.000	0.000	0.000	0.000	0.000
Optimal cutoffs	86.900	24.7859	0.567	4.711	8.380	4.697	37.406	1.309	113.022	8.624	216.218	763.525	4.924	36.196
J-Youden	0.224	0.170	0.257	0.177	0.148	0.257	0.234	0.191	0.293	0.179	0.217	0.246	0.261	0.207
Sensitivity (%)	58.50	48.20	60.40	55.80	55.20	60.40	58.30	56.80	59.60	62.90	52.70	56.50	61.60	56.80
Specificity (%)	63.90	68.80	65.30	61.90	59.60	65.30	65.10	62.30	69.70	55.00	69.00	68.10	64.50	63.90

ROC is used to compare the evaluation ability of various indicators in different sexes for hypertension

*WC* Waist circumference, *BMI* Body mass index, *WHtR* Waist height ratio, *VAI* Visceral adiposity index, *ABSI* A body shape index, *BRI* Body roundness index, *LAP* Lipid accumulation product, *CVAI* Chinese visceral adiposity index, *CI* Conicity index, *TyG* Triglyceride glucose, *TyG-BMI* TyG related to BMI, *TyG-WC* TyG related to WC, *TyG-WHtR* TyG related to WHtR, *METS-IR* Metabolic score for insulin resistance

## Discussion

According to the 2018 Chinese Guidelines for Prevention and Treatment of Hypertension [17], this research predicts the risk index of hypertension in middle-aged and elderly people in China by the 14 obesity-related indexes. It is a novel one. We know that before this, no previous research paper has explored the most closely related indicators of hypertension by comparing 14 obesity and lipid-related indicators. This study included 9488 male and female participants over 45 years old. Among the 9488 subjects, the proportion of hypertension was 41.15%. Among the 4354 males, 1742(40.01%) and 5134 females, 2162(42.11%) suffered from hypertension. Based on the entire country’s investigation [2], the prevalence of high blood pressure among adults in China is about 23.2%, among which the prevalence of hypertension increases with age, the prevalence of hypertension in the population aged 65 and over is over 55%, and the prevalence rate in men is higher than that in women. Therefore, it is necessary to explore the correlation between obesity and lipid-related index and hypertension, screen high-risk groups of the disease, and carry out individualized health care measures.

Table 3 shows that the ABSI is significantly related to hypertension in men, after adjusting the individual characteristics, but it is not statistically significant in women. The data in Table 4 show that ABSI is associated with hypertension in middle-aged and elderly people in our country, and the degree of association is much lower than the other 13 indices. Because the deviation of ABSI from its average value is low, the association between ABSI and hypertension was not significant [31]. ROC analysis of obesity- and lipid-related indices showed that the AUC of all indexes of male and female was statistically meaningful (*P* < 0.05). Among the 14 indexes, CVAI, WHtR, BRI, and TyG -WHtR have received special attention.

A study conducted by Fiorentino, T.V. [32] to test the risk factors related to the progression of hypertension in patients with prehypertension showed that VAI was an independent risk factor for the progression of hypertension. However, the significant overlap of VAI confidence intervals does not imply that VAI is superior to other measures of obesity in terms of how closely it is associated with hypertension. Notably, the combination of VAI and WC showed the closest indicator of hypertension. This conclusion is consistent with the results of a cohort study [33] conducted in Chengdu, Sichuan Province, China. It can be analyzed for the following reasons: the difference in body fat distribution between different races is also obvious. According to the distribution law of body fat in the Asian population, Xia, M. F. put forward the index of CVAI based on VAI to calculate the visceral fat area of China people [34]. In this study, compared with VAI, CVAI is more closely associated with hypertension in Chinese middle-aged and elderly people.

A cohort study [35] of 10,304 Chinese adult residents showed that CVAI outperformed other measures of visceral obesity in predicting the incidence of hypertension in either men or women. Similarly, Lin, M [36], in a 2022 cohort of 2033 participants, showed that CVAI performed best in predicting hypertension. In this study, ROC analysis was performed on obesity-and lipid-related indicators, maximum AUC (male: AUC = 0.660, 95% CI = 0.643–0.676 and optimal cut-off = 111.142, female: AUC = 0.699, 95% CI = 0.685–0.713, and optimal cut-off = 113.022) was observed for the CVAI. CVAI is most closely related to hypertension in middle-aged and elderly Chinese. This conclusion is consistent with the conclusions of previous studies.

In this study, WHtR and BRI were closely related indicators of hypertension, and the relationship is second only to CVAI. Lee, J. W’s [37] study showed that WHtR and WC were superior to BMI as screening tools in the incidence of hypertension in middle-aged and elderly Koreans. At the same time, A prospective cohort study [38] of 812 participants showed that WHtR had a significant advantage over WC, and BMI in screening people at high risk for hypertension. These studies are consistent with the view of this paper that WHtR is closely related to hypertension.

In this study, the association strength between four lipid indexes, such as TyG index, TyG- body mass index, TyG-WC and TyG-WHtR, and hypertension was evaluated. There is a significant relationship between TyG-related factors and hypertension [39, 40]. In this study, WHtR (AUC = 0.651, 95% CI = 0.635–0.668) and TyG-WHtR (AUC = 0.651, 95%CI = 0.634–0.667) were associated with hypertension to approximately the same extent. But in women, the relevance of TyG-WHtR (AUC = 0.674, 95%CI = 0.660–0.689) was superior to WHtR (AUC = 0.664, 95%CI = 0.649–0.679). Therefore, TyG-WHtR is more closely associated with hypertension than WHtR in the middle-aged and elderly Chinese population.

BRI offers the possibility to screen people at high risk of hypertension. Chang, Y’s study [41] of two new measures to identify high blood pressure reported a low association with ABSI and the strongest association with BRI. A study [42] that predicted risk factors for cardiovascular disease in China reported that BRI was superior to other indicators in its predictive power. In this study, it should be noted that BRI and WHTR showed the same association in both males and females.

Risk factors for obesity and fat-induced hypertension are broad and include various adipokines, abnormal gut microbiota, sympathetic nervous system activation, excessive vasopressin, natriuretic hormone deficiency, and vascular and renal dysfunction [43–45]. In this study, obesity and lipid related indicators were used to assess individual physical status. Among the 14 indicators, CVAI showed the best ability to evaluate hypertension. At the same time, CVAI calculation only requires HDL-C, TG and basic body measurement data, which is easy to be met in clinical work. Middle-aged and elderly Chinese are at high risk of hypertension, and hypertension is also a high-risk factor for other cardiovascular diseases. CVAI can be used to quickly assess the risk of hypertension, screen high-risk groups of hypertensions, and take health management measures to prevent hypertension. However, HDL-C, and TG are invasive procedures that are often not readily available in primary health care clinics. For middle-aged and older patients aged ≥ 45 years visiting primary health care clinics, WHtR and BRI are more likely to address such questions. These two measures can be used to screen people at high risk of hypertension with only WC and Height. It is worth noting that WHtR is easier to calculate than BRI.

## Strengths and limitations of the study

This study has several advantages. The data of this cross-sectional study were obtained from a national survey of middle-aged and elderly community residents, with a wide range of sample sources and an adequate sample size. Previous studies only explored the correlation between one or several different indicators and hypertension, while this study explored the relationship between 14 different obesity and lipid indicators and hypertension from different directions.

The study has several limitations. Due to the differences in diet, activity, and ethnic characteristics between the Chinese population and other countries, the results of this study are only applicable to the population of various regions in China, and data cannot be easily transmitted to other populations. In this study, uric acid and insulin resistance were included in the risk analysis model, and more clinical and laboratory parameters need to be included. Most of the obesity and lipid indicators are not used in clinical practice, and further clinical studies are needed to examine the clinical benefits of the indicators. This is a cross-sectional study, and the predictive power of this study is insufficient. Further cohort studies are needed to identify the best predictors of hypertension in China.

## Conclusions

After adjusting for covariate factors, most obesity and lipid-related factors were independently associated with hypertension and presented as risk factors. CVAI has the best ability to screen people at high risk of hypertension, and relevant conditions are more readily available in large hospitals. For primary health care clinics, WHtR is recommended for assessment, which only requires simple physical measurement and calculation. Therefore, it is recommended to use CVAI and WHtR for hypertension risk assessment in middle-aged and elderly people aged ≥ 45 years and timely health interventions to reduce the incidence of hypertension.

### Supplementary Information

**Additional file 1: Supplemental Table 1.** Adjusted covariates in statistical models and their classification.

## Data Availability

Data can be accessed via http://charls.pku.edu.cn/.

## Abbreviations

HTN: Hypertension
BMI: Body mass index
WC: Waist circumference
WHtR: Waist-height ratio
CI: Conicity index
VAI: Visceral adiposity index
CVAI: Chinese visceral adiposity index
LAP: Lipid accumulation product
ABSI: A body shape index
BRI: Body roundness index
TyG: Triglyceride glucose
TyG-BMI: Triglyceride glucose-body mass
TyG-WC: Triglyceride glucose-waist circumference
TyG-WHtR: Triglyceride glucose-waist-height ratio
ROC: Receiver operator curve
AUC: Area under curve
TG: Triglyceride
HDL-C: High-density lipoprotein
BP: Blood pressure
FPG: Fasting plasma glucose blood glucose
CHARLS: China Health and Retirement Longitudinal Study
CAPI: Computer-aided personal interview
OR: Odds ratio
CVD: Cardiovascular disease
SUA: Serum acid
Mets-IR: Metallic score for internal resistance

## References

[1] Liu LS (2011). 2010 Chinese guidelines for the management of hypertension. Zhonghua Xin Xue Guan Bing Za Zhi.

[2] Wang Z (2018). Status of hypertension in China: results from the China hypertension survey, 2012–2015. Circulation.

[3] Oliveros E (2020). Hypertension in older adults: assessment, management, and challenges. Clin Cardiol.

[4] Stanaway JD, Afshin A, Gakidou E (2018). Global, regional, and national comparative risk assessment of 84 behavioural, environmental and occupational, and metabolic risks or clusters of risks for 195 countries and territories, 1990-2017: a systematic analysis for the Global Burden of Disease Study 2017. Lancet.

[5] Li J (2017). Urban-rural disparities in hypertension prevalence, detection, and medication use among Chinese Adults from 1993 to 2011. Int J Equity Health.

[6] Chen WW (2017). China cardiovascular diseases report 2015: a summary. J Geriatr Cardiol.

[7] Wang Q (2018). Rural-urban difference in blood pressure measurement frequency among elderly with hypertension: a cross-sectional study in Shandong, China. J Health Popul Nutr.

[8] Ding L (2020). Smoking, heavy drinking, physical inactivity, and obesity among middle-aged and older adults in China: cross-sectional findings from the baseline survey of CHARLS 2011–2012. BMC Public Health.

[9] You Y (2018). Hypertension and physical activity in middle-aged and older adults in China. Sci Rep.

[10] Sowers JR (2013). Diabetes mellitus and vascular disease. Hypertension.

[11] da Silva AA (2020). Role of hyperinsulinemia and insulin resistance in hypertension: metabolic syndrome revisited. Can J Cardiol.

[12] Masuo K (2003). Serum uric acid and plasma norepinephrine concentrations predict subsequent weight gain and blood pressure elevation. Hypertension.

[13] Saito Y (2021). Uric acid and cardiovascular disease: a clinical review. J Cardiol.

[14] Tao LC (2022). Triglyceride-glucose index as a marker in cardiovascular diseases: landscape and limitations. Cardiovasc Diabetol.

[15] Lin YP (2023). Insulin resistance indices and carotid intima-media thickness in physically fit adults: CHIEF atherosclerosis study. Endocr Metab Immune Disord Drug Targets.

[16] Koliaki C, Liatis S, Kokkinos A (2019). Obesity and cardiovascular disease: revisiting an old relationship. Metabolism.

[17] Liu LS, Wu ZS, Wang JG (2019). 2018 Chinese guidelines for prevention and treatment of hypertension-a report of the revision committee of Chinese guidelines for prevention and treatment of hypertension. J Geriatr Cardiol.

[18] Zhang L (2022). Mediator or moderator? The role of obesity in the association between age at menarche and blood pressure in middle-aged and elderly Chinese: a population-based cross-sectional study. BMJ Open.

[19] Khanna D (2022). Body mass index (BMI): a screening tool analysis. Cureus.

[20] Qian JD (2022). Comparative analysis of the association between traditional and lipid-related obesity indicators and isolated systolic hypertension: association of obesity indicators with ISH. BMC Cardiovasc Disord.

[21] Vicente-Herrero MT (2023). Visceral adiposity index (VAI) and dysfunctional adiposity index (DAI). Relationship with obesity parameters. Semergen.

[22] Calderón-García JF (2021). Effectiveness of Body Roundness Index (BRI) and a Body Shape Index (ABSI) in predicting hypertension: a systematic review and meta-analysis of observational studies. Int J Environ Res Public Health.

[23] Gao W (2023). The association between the body roundness index and the risk of colorectal cancer: a cross-sectional study. Lipids Health Dis.

[24] Li H (2022). The lipid accumulation product is a powerful tool to diagnose metabolic dysfunction-associated fatty liver disease in the United States adults. Front Endocrinol (Lausanne).

[25] Zhang A (2022). Conicity-index predicts all-cause mortality in Chinese older people: a 10-year community follow-up. BMC Geriatr.

[26] Chen X (2022). Associations between abdominal obesity indices and nonalcoholic fatty liver disease: Chinese visceral adiposity index. Front Endocrinol (Lausanne).

[27] Li H (2022). Triglyceride-glucose index variability and incident cardiovascular disease: a prospective cohort study. Cardiovasc Diabetol.

[28] Selvi NMK (2021). Association of triglyceride-glucose index (TyG index) with HbA1c and insulin resistance in type 2 diabetes mellitus. Maedica (Bucur).

[29] Jiang C (2021). Triglyceride glucose-body mass index in identifying high-risk groups of pre-diabetes. Lipids Health Dis.

[30] Bello-Chavolla OY (2018). METS-IR, a novel score to evaluate insulin sensitivity, is predictive of visceral adiposity and incident type 2 diabetes. Eur J Endocrinol.

[31] Ji M, Zhang S, An R (2018). Effectiveness of A Body Shape Index (ABSI) in predicting chronic diseases and mortality: a systematic review and meta-analysis. Obes Rev.

[32] Fiorentino TV (2018). Visceral adiposity index (VAI), a powerful predictor of incident hypertension in prehypertensives. Intern Emerg Med.

[33] Zhang Z (2018). Visceral adiposity index (VAI), a powerful predictor of incident hypertension in prehypertensives. Intern Emerg Med.

[34] Xia MF (2016). A indicator of visceral adipose dysfunction to evaluate metabolic health in adult Chinese. Sci Rep.

[35] Han M (2021). Chinese visceral adiposity index, a novel indicator of visceral obesity for assessing the risk of incident hypertension in a prospective cohort study. Br J Nutr.

[36] Lin M (2022). Chinese visceral adiposity index is associated with incident renal damage in patients with hypertension and abnormal glucose metabolism: a longitudinal study. Front Endocrinol (Lausanne).

[37] Lee JW (2015). Anthropometric indices as predictors of hypertension among men and women aged 40–69 years in the Korean population: the Korean Genome and Epidemiology Study. BMC Public Health.

[38] Wang Q (2018). Anthropometric indices predict the development of hypertension in normotensive and pre-hypertensive middle-aged women in Tianjin, China: a prospective cohort study. Med Sci Monit.

[39] Wang K (2021). Association of triglyceride-glucose index and its interaction with obesity on hypertension risk in Chinese: a population-based study. J Hum Hypertens.

[40] Mao Y (2022). Association of three insulin resistance indices with hypertension and body weight among Uyghur adults in rural areas of Xinjiang, China. J Clin Hypertens (Greenwich).

[41] Chang Y (2016). The feasibility of two new anthropometric indices to identify hypertension in rural China: a cross-sectional study. Medicine (Baltimore).

[42] Li Y (2022). Body roundness index and waist-hip ratio result in better cardiovascular disease risk stratification: results from a large Chinese cross-sectional study. Front Nutr.

[43] DeMarco VG, Aroor AR, Sowers JR (2014). The pathophysiology of hypertension in patients with obesity. Nat Rev Endocrinol.

[44] Hall JE (2019). Obesity, kidney dysfunction and hypertension: mechanistic links. Nat Rev Nephrol.

[45] Jordan J (2018). Natriuretic peptides in cardiovascular and metabolic crosstalk: implications for hypertension management. Hypertension.

