# Supplementary material for: Obesity-and lipid-related indices as a risk factor of hypertension in mid-aged and elderly Chinese: a cross-sectional study
Source: BMC Geriatr. 2024 Jan 20;24:77. doi: 10.1186/s12877-023-04650-2 (PMC10800050; doi:10.1186/s12877-023-04650-2)
Supplement: Supplementary file 1 — Additional file 1: Supplemental Table 1. Adjusted covariates in statistical models and their classification. [file 12877_2023_4650_MOESM1_ESM.docx]

**Supplemental Table 1. Adjusted covariates in statistical models and their classification**

| Covariates | Classification |
| --- | --- |
| Age(years) | 1) below 45-54  2) 55-64  3) 65-74  4) above 75 |
| Education level | 1) illiterate  2) less than elementary school  3) high school  4) above vocational school |
| Marital status | 1) the single (divorced, and never married, widowed, or separated)  2) married |
| Current residence | 1) rural  2) urban |
| Current smoking | 1) current smokers  2) former smokers  3) never smokers |
| Alcohol drinking | 1) never drinker  2) less than once a month  3) more than once a month |
| Taking activities | 1) no  2) yes |
| Having regular exercises | 1) no physical exercise  2) less than regular physical exercises  3) regular physical exercises |
| Chronic diseases(counts) | 1) 0  2) 1-3  3) 4-14 |

Chronic diseases include hypertension, dyslipidemia, diabetes or high blood sugar, cancer or malignancy, chronic lung disease, liver disease, heart disease, stroke, kidney disease, digestive system diseases, emotional, neurological or psychiatric conditions, memory-related diseases, arthritis or rheumatic diseases, and asthma.
